# Adaptations to an implementation study for integrating hypertension management into HIV care in Lagos, Nigeria: application of the FRAME

**DOI:** 10.1186/s43058-026-00869-3

**Published:** 2026-03-10

**Authors:** Chioma Hope Nwankwo, Oluwayemi Dorcas Odejobi, Oluwatosin Olaseni Odubela, Shivani Mishra, Deborah Onakomaiya, Nafesa Kanneh, Ucheoma Nwasozuru, Aina Olufemi Odusola, Weixi Chen, Aderonke Bayonle, Ifeoma Idigbe, David Oladele, Bamidele Olusegun Tayo, Jiyuan Hu, Zaidat Musa, Angela A. Aifah, Gbenga Ogedegbe, Juliet Iwelunmor, Oliver Ezechi

**Affiliations:** 1 Clinical Sciences Department, Nigerian Institute of Medical Research, Lagos, Nigeria; 2 Institute for Excellence in Health Equity, NYU Grossman School of Medicine, New York, NY USA; 3 Vilcek Institute of Graduate Biomedical Sciences, NYU Grossman School of Medicine, New York, NY USA; 4 Department of Population Health, NYU Grossman School of Medicine, New York, NY USA; 5 Department of Implementation Science, Division of Public Health Sciences, Wake Forest University School of Medicine, Winston-Salem, NC USA; 6 Department of Community Health and Primary Health Care, Lagos State University Teaching Hospital, Lagos, Nigeria; 7 Loyola University Chicago, Maywood, IL USA; 8 Monitoring and Evaluation Unit, Nigerian Institute of Medical Research, Lagos, Nigeria; 9 Division of Infectious Diseases, Washington University School of Medicine, Saint Louis, MO USA

**Keywords:** Hypertension, HIV, Lagos, FRAME, Adaptations

## Abstract

**Background:**

Implementation strategies are dynamic and multi-faceted, and may require adaptations to fit implementation contexts, especially in lower-and-middle income countries. We report the adaptations for an ongoing late-stage implementation science trial (R01HL147811) that integrates hypertension management into HIV care in Lagos, Nigeria – a country with a high dual-disease burden – through the Task Strengthening Strategy for Hypertension (TASSH) intervention and Practice Facilitation implementation strategy.

**Methods:**

FRAME (Framework for Reporting Adaptations and Modifications—Enhanced) modules were used to record adaptations to the intervention (i.e., TASSH) respectively, enhance participant recruitment and retention rates, and increase frequency of trainings. Data collection sources included (not limited to) patient records, nurses’ logs, and minutes of implementation review meetings. Data across these sources was coded retrospectively by trained research staff and triangulated during virtual meeting discussions. Once consensus was reached, data was mapped onto the relevant framework modules using Microsoft Excel.

**Results:**

We modified FRAME to include an additional component on ‘what was originally planned’ for the context of the adaptations. There were twelve adaptations identified during the implementation of the study. The adaptations characterized by using the frameworks included reordering recruitment start dates of study cohorts, providing patients incentives to attend follow-up visits, adding feeder sites to the study sites, and increasing the frequency of training to account for the high nurse turnover in the primary healthcare centers. Overall, 25% of the adaptations involved expanding the structure of the intervention and implementation strategies, and 33% involved adding new elements to the strategies. All adaptations occurred in the implementation phase of the trial.

**Conclusion:**

Based on our experiences, the characterization of the adaptations using FRAME demonstrates their combined applicability to an ongoing trial that can be tailored to fit the local context.

**Trial registration:**

ClinicalTrials.gov ( NCT04704336). Registered on 11 January 2021.

**Supplementary Information:**

The online version contains supplementary material available at 10.1186/s43058-026-00869-3.

Contributions to the literature

- We provide new evidence on improving the effectiveness of task shifting and practice facilitation in the management of hypertension in the HIV clinics.
- We provide critical insights for scaling integrated HIV and hypertension care models, emphasizing the importance of context-specific adaptations to an ongoing trial for sustainability in low-resource settings.
- This is the first study to apply the FRAME model to document adaptations in integrating hypertension and HIV care in West Africa, expanding its use in low-resource contexts.

## Background

HIV management and care remain a pressing concern, along with the rising prevalence of hypertension among people living with HIV (PLHIV), which adds another layer of health complications [1, 2]. Nigeria, in particular, accounts for the highest population of PLHIV in West Africa, with a 13 to 50% prevalence of hypertension in this cohort [1]^.^ Since 2017, research has continued to indicate a rising prevalence of hypertension among PLHIV in the country, highlighting the urgent need for integrated management strategies to mitigate this dual disease burden [1, 3, 4]. Addressing this dual burden requires integrated healthcare approaches that target the unique needs of individuals living with both conditions, ensuring comprehensive and sustainable care strategies for better health outcomes. Primary healthcare centers (PHCs) can serve as a point of care for implementing these integrated approaches.

### Task-strengthening strategy for hypertension control [TASSH] for integrated Hypertension and HIV care

PHCs are the smallest and closest units of public healthcare facilities in Nigerian communities. However, the services delivered at PHCs are limited due to a shortage of healthcare workers, exacerbating the challenges faced by the healthcare system [5]. To address the shortage of healthcare workers, task-shifting has emerged as a strategic approach. This involves delegating certain tasks from highly specialized healthcare workers (HCWs) to less specialized HCWs, thereby optimizing resource utilization [5]. The Lagos State's Ministry of Health task-shifting policy embodies this approach and plays a pivotal role in healthcare delivery within the state [4]. Previous programs and studies in Lagos have demonstrated the effectiveness of task-shifting, particularly in the management of diarrhea, upper respiratory tract infections, and antenatal care in PHCs [6–8].

**Fig. 1:**
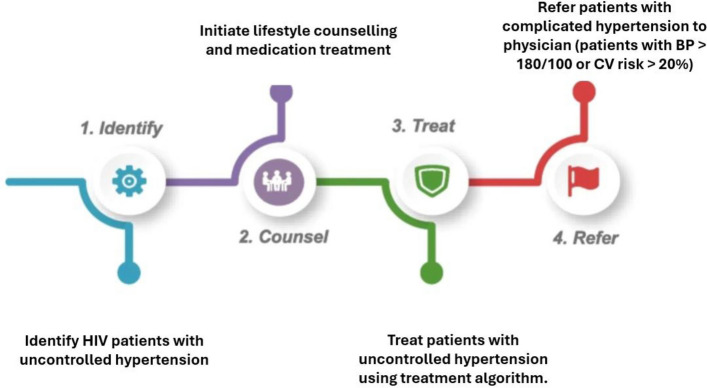
Description of the 4 components of TASSH: Identify, Counsel, Treat and Refer (ICTR) that were used by the nurses to perform the ICTR for PLWH with uncontrolled, uncomplicated hypertension (as seen in, and figure obtained from Aifah et al.) [9]

Building on this foundation, the National Institutes of Health (NIH) – sponsored study [TASSH – Task Strengthening Strategy for Hypertension control, NIH study number: R01HL147811] employs a Late Stage T4 Implementation Science design to evaluate the effectiveness of Practice Facilitation on the integration, adoption, and sustainability of the TASSH intervention within PHCs in Lagos State, Nigeria [9]. This study recognizes the dynamic nature of implementation contexts, allowing for ongoing adaptations to maintain relevance and effectiveness in real-world settings. Figure 1 provides an overview of the TASSH intervention, which includes *identifying, counseling, treating, and referring* (ICTR) PLIVH with co-morbid hypertension. Additional details on the TASSH intervention and implementation are available in the study protocol [9].

### Adaptations to implementation strategy and interventions in late-stage clinical trials

The growing body of evidence underscores the importance of tailoring evidence-based interventions to the specific contexts in which they are implemented, particularly in clinical trials [10]. Documenting these adaptations, both for the interventions and the strategies used to implement them, is essential for ensuring transparency and upholding scientific integrity. Such documentation not only aids researchers in understanding how interventions are modified in real-world settings but also plays a critical role in interpreting study outcomes, thereby maintaining the validity and credibility of clinical research findings [9, 10]. Specifically, documenting adaptations in trials that integrate HIV and hypertension interventions in Nigeria helps to replicate successful strategies and informs the design of future trials in settings with complex or multi-tiered health systems. This approach ultimately enhances the effectiveness and relevance of healthcare interventions [9].

### The FRAME approach to document adaptations

The Framework for Reporting Adaptations and Modifications for Evidence-Based Interventions (FRAME) model proposed by Stirman and colleagues captures modifications and adaptations made to an intervention [10]. FRAME offers a structured approach to documenting general adaptations and modifications, enhancing the accuracy and comprehensiveness of reporting [10]. The framework is particularly valuable for documenting complex and multifaceted interventions. This is crucial for trials that integrate multiple care components, like hypertension management within an HIV care continuum. By leveraging the FRAME model, researchers can accurately capture and articulate the nuances of adaptations, thereby enhancing the implementation and comprehension of integrated programs like TASSH [11, 12]. The components of FRAME and how it is used to document adaptations and modifications of an intervention is depicted in Stirman and colleagues’ original work [10].

A growing number of studies have used and/or proposed the use of FRAME to document general adaptations and modifications [13–16]. These studies are limited by a focus on mental health interventions in high-income countries [15, 16]. Consequently, there has been limited exploration of how FRAME operates in low-resource settings. The applicability and effectiveness of FRAME in capturing adaptations within these contexts remain underexplored, creating a gap in understanding how such frameworks function within the complexity of multifaceted strategies like practice facilitation and the TASSH intervention in low- and middle-income countries (LMICs). To address this limitation in tracking adaptations, we aim to a) characterize the adaptations made to a multi-faceted late-stage trial that uses practice facilitation and TASSH and b) share the implications of the adaptations on the overall trial.

## Methods

### Study setting

#### Overview of partnerships in the study

The study is a collaboration between NYU Grossman School of Medicine (NYU), New York, USA, Washington University in St. Louis (WashU), Missouri, USA, and Nigerian Institute of Medical Research (NIMR), Lagos, Nigeria. NIMR serves as the operational hub, with further collaborations with state actors; Lagos State Primary HealthCare Board (LSPHCB) and Lagos State AIDS Control Agency (LSACA), to ensure appropriate oversight and conduct of the study.

The Nigerian Institute of Medical Research (NIMR) is the foremost health research institute in the country with a mandate to conduct research on diseases of public health importance [17]. The institute operates an antiretroviral therapy (ART) clinic, which has been in existence since 2002. The ART clinic has cumulatively enrolled over 25,000 PLHIV [17, 18]. The institute has a good working relationship with LSPHCB and LSACA, as well as contributes to the National Treatment Taskforce Committee on the care and management of HIV/AIDS in the country. Notably, LSACA is the agency that addresses the care and management of HIV in Lagos state. The agency (LSACA) provided the number of PLHIV (currently in care) in each PHC, while LSPHCB provided access to the nurses/Community Health Officers (CHOs) within the PHCs.

#### Overview of the TASSH trial

The study was conducted in PHCs across Lagos State that provide comprehensive HIV care and management. Sixty-seven (67) PHCs provide comprehensive HIV care and management in Lagos State at the onset of the study. Using cluster randomized controlled trial methods, 30 PHCs were selected, representing fifteen local government areas (LGAs) within the state. Each cohort, consisting of six PHCs, was onboarded into the study and randomized to two arms that receives PF intervention or Self-directed control conditions. The selected PHCs were then randomized to receive the TASSH intervention. Within each cohort, three PHCs were assigned to receive practice facilitation, while the remaining three continued with standard care.

The primary objective of the PHC is to provide preventive care and services that are affordable and accessible to people and communities. These activities include immunization, family planning, maternal and child health services, cervical cancer screening, perinatal care, health education and counseling, treatment of minor ailments (uncomplicated malaria, diarrheal illness, pneumonia), community outreach, and care of wounds following trauma.

At these PHCs, PLHIV engage with various healthcare professionals, including nurses, CHOs, pharmacy technicians, midwives (especially during delivery), and phlebotomists. Occasionally, PLHIV also interacts with doctors to address treatment failures (clinical, virological, or immunological) or treatment of other co-morbidities beyond tuberculosis. Thus, the PHCs are crucial in mitigating risks associated with chronic and lifestyle-related conditions. They play a key role in the early detection, assessment, management of chronic diseases (such as hypertension), and support individuals in taking proactive control of their health.

Due to a shortage of clinicians (which can be attributed to them seeking opportunities abroad) [19], the Lagos State government implemented a task-shifting policy to allow other HCWs to provide services typically provided by clinicians [19]. Building on this policy, the TASSH study aims to strengthen and expand task-shifting by enhancing nurses’ and CHOs’ skills to manage co-morbid hypertension among PLHIV in PHCs across Lagos State.

### Study population

A total number of 960 study participants receiving care and management for HIV at the selected PHCs were planned to be recruited. PLHIV who were 18 years and above with uncontrolled blood pressure *(systolic blood pressure of 140–179 mmHg and/or diastolic blood pressure of 90–100 mmHg)* were eligible for study inclusion. The exclusion criteria included study participants with blood pressure readings beyond the above definition for uncontrolled hypertension, prior history of co-morbidities (stroke, chronic kidney disease, diabetes mellitus, transient ischemic attack, liver disease), ongoing pregnancy, lactating mothers, and inability to provide informed consent or refusal to participate in the study.

### Study design

The Framework for Reporting Adaptations and Modifications—Enhanced (FRAME) model was used to document adaptations of the TASSH intervention in Lagos state. A Microsoft Excel spreadsheet was designed to document challenges and adaptations encountered during the study while using appropriate FRAME modules to critically evaluate the adaptations. This was achieved by employing mixed methods (interviews, review meetings, and documentations). Detailed explanations of the FRAME modules have been previously published [10].

### Data collection

In response to lessons learnt during the early stages of the study, the team identified unforeseen gaps. Strategies and potential adaptations were developed during study review meetings. Additionally, interactions with HCWs (at both administrative and clinical levels), practice facilitators, implementation partners, and study steering committee members were also documented.

### Study team meetings

During the implementation phase of the study, the team, consisting of principal investigators (PIs), co-investigators (Co-Is), study coordinators, data managers, and study lead nurses, held monthly meetings to review study progress, discuss challenges, and address barriers. Additionally, study coordinators and the implementation team convened bi-weekly, while the data management team met once a month. Structured notes from these meetings captured action steps, attendance, and deliverables. These notes were central to discussions among the PIs, where necessary adaptations to the study were made and approved.

### Narrative reports

The study implementation team conducted site visits to the PHCs to observe how HCWs were executing the TASSH intervention at their facilities. Additionally, practice facilitators made monthly visits to the PHCs within their jurisdiction and provided feedback to the implementation team using dedicated fidelity tools. The observations and feedback collected from these site visits were documented and played a key role in informing subsequent adaptations to the intervention.

Reporting guide follows the SQUIRE 2.0 reporting guidelines, included as an additional file 1.

## Results

Between November 2021 and April 2023, twelve adaptations were made to refine the TASSH intervention. These adjustments, collaboratively decided by the study team during the implementation phase, reflect a proactive approach to optimizing participant recruitment, enrollment, and retention while ensuring high-quality intervention delivery. Each adaptation addressed specific challenges encountered during the study and aimed to enhance the overall effectiveness and efficiency of the intervention.

The adaptations were categorized into three primary goals:

A. Increasing Participant Recruitment and Enrollment: Strategies were implemented to attract and enroll more participants.
B. Enhancing Patient Follow-Up and Retention Rates: Measures were taken to improve the retention of participants throughout the study.
C. Ensuring Capacity Building and Quality Intervention Delivery: Efforts were made to build capacity, maintain high standards of intervention delivery, and uphold implementation fidelity.

**Fig. 2:**
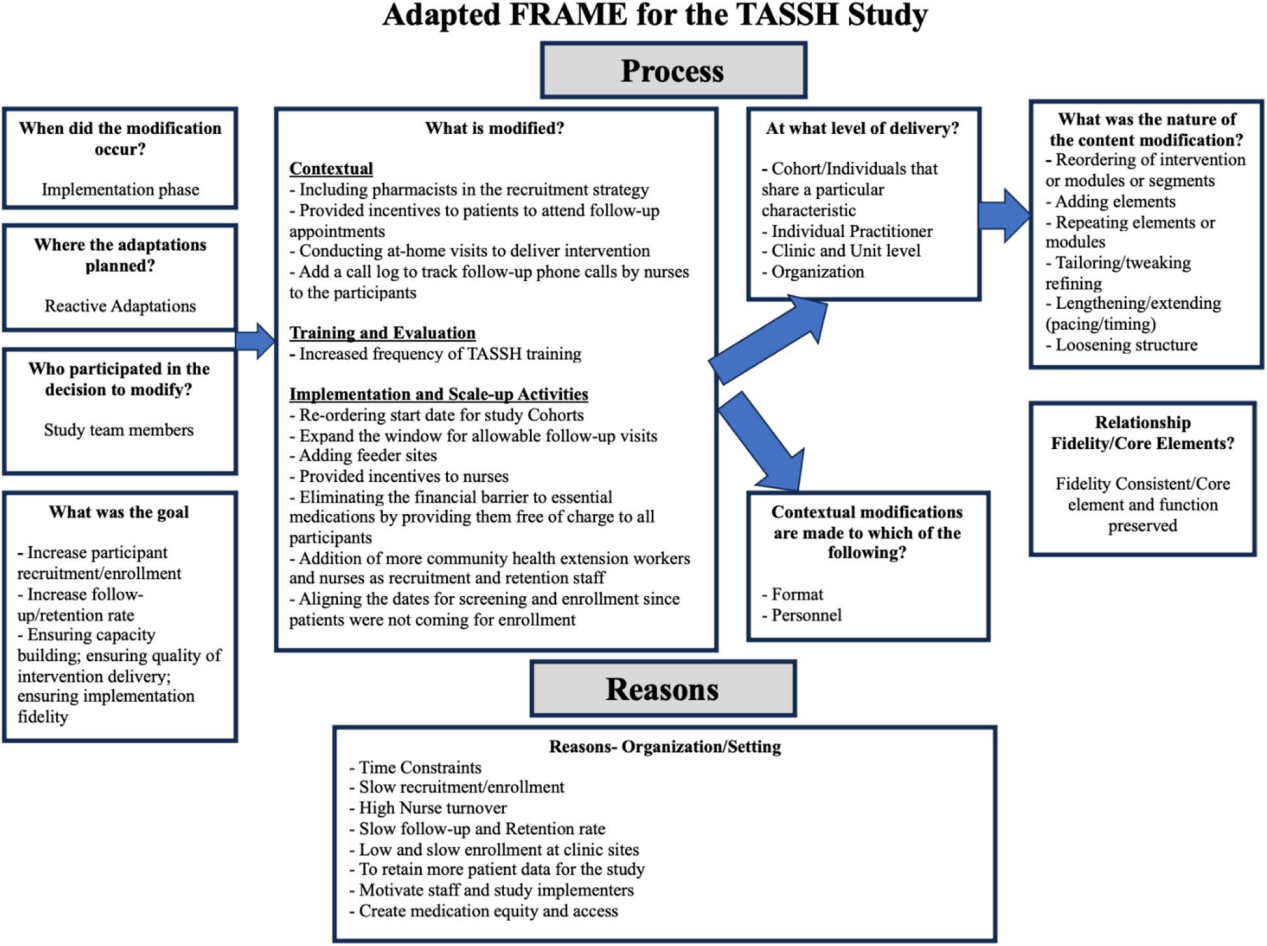
Adapted FRAME for documenting general adaptations, in the TASSH Intervention

Figure 2 illustrates an adapted version of the FRAME model, tailored to reflect the adaptations made to the TASSH intervention. It provides a detailed view of the adaptation process and the rationale behind each change, following the framework proposed by *Stirman *et al*.* [10]

### Adaptations

#### Increasing participant recruitment/enrollment

##### Re-ordering start date for study cohorts

This adaptation sought to optimize the study's efficacy by reconfiguring the commencement schedule for Cohorts 2 to 5 of the TASSH intervention. The rationale behind this modification was to strategically prioritize cohorts with larger sites, enabling them to initiate the study sooner. This re-ordering ensured that Cohorts 2 and 3, (with a higher number of PLHIVs receiving care at the PHCs) commenced the trial earlier, while Cohorts 4 and 5, characterized by a smaller number of PLHIVs, began involvement at a later stage. Originally, the sequencing of cohorts was not contingent on their size; however, the need to align with the NIH Accrual Plan and address recruitment challenges prompted this strategic alteration. By realigning the start date of the cohort, the study aimed to bolster recruitment numbers and achieve a more representative and robust sample size.

##### Integrating pharmacists in recruitment strategy

This modification marked a significant enhancement in the recruitment strategy by integrating pharmacists working at the PHCs. Recognizing that PLHIV receiving treatment at participating PHCs predominantly engaged with pharmacists during Antiretroviral drug pick-ups visits rather than clinic nurses, this modification strategically leveraged pharmacists as key allies in participant recruitment. Pharmacists underwent specialized training to identify eligible participants and refer them to TASSH study nurses/CHOs at the clinic. This proactive modification was prompted by the challenge of slow recruitment and enrollment, attributed to patients' frequent interactions with pharmacists during drug pickups (every 2, 3, or 6 months) as opposed to clinic appointments (every 6 months). The modification aimed to boost participant enrollment and recruitment rates, underscoring a dynamic and proactive approach to optimize recruitment channels and maximize the study's impact and effectiveness.

##### Introducing feeder sites

Recognizing the challenge of low enrollment rates, the study expanded its reach by including patients from satellite PHCs to bolster enrollment. These sites were enlisted to boost enrollment through referrals, despite not being formally part of the study. Initially, the enrollment strategy solely targeted patients from the study PHCs, overlooking the potential contributions of non-study PHCs. However, based on insights and recommendations from the study's Data and Safety Monitoring Board (DSMB) and local stakeholders, it became evident that excluding smaller facilities might have hindered enrollment progress. Consequently, during the implementation phase, adjustments were made to refine the enrollment strategy. This involved customizing and enhancing the implementation and scale-up strategy by integrating additional feeder sites to optimize enrollment efforts. By leveraging these satellite sites for patient referrals, the study tapped into a broader patient demographic, ultimately enriching the dataset and advancing its objectives.

##### Nurse incentives

Nurses at PHCs received incentives to address challenges related to their motivation and engagement. This involved offering incentives such as phone data cards and certifications to boost their motivation. The aim was to use motivation as a strategy to actively engage nurses, thereby improving both enrollment efforts and retention rates for the study. Initially, nurses were not incentivized to conduct the components of the TASSH Intervention (5As) for study participants (PLHIV) with comorbid hypertension, as these interventions were considered part of routine care in the PHCs. However, recognizing the need to incentivize nurses due to competing interests and activities at the PHCs, as well as to secure their commitment and motivation to the study, incentives were introduced. The overall goal of this modification was to increase enrollment by enhancing staff motivation and dedication to the study, thus creating a more conducive environment for participant engagement and retention.

##### Extending provision of free medication

The TASSH study introduced an initiative to extend the provision of free medication, aiming to tackle medication access and equity issues. This involved supplying anti-hypertensive medications to every study participant, regardless of their socio-economic status. Initially, medications was only provided free of charge to individuals who self-identified as disadvantaged and unable to afford it (e.g. indigenous groups). However, recognizing the importance of ensuring medication equity and access for all participants, this modification was made during the implementation phase. The adjustment aimed to create a more inclusive approach to medication access. Ultimately, the goal of this modification was to boost enrollment and retention rates by guaranteeing that all participants had equal access to necessary anti-hypertensive medication, thereby promoting health equity within the study population.

##### Increasing community health officers and nurses for recruitment and retention

To address challenges in enrollment and retention, the study implemented the addition of more CHOs and Nurses as recruitment and retention staff. While CHOs and nurses were recruited as intended initially, the rapid expansion of the study across Lagos necessitated additional personnel to ensure effective coverage. This modification involved increasing the number of recruitment and retention staff to enhance the original structure. The rationale behind this change stemmed from low and slow enrollment rates attributed to staff shortages, particularly affecting patient follow-up in cohort 3. The goal of this modification was to enhance enrollment by strengthening recruitment and retention efforts through the addition of more personnel. This would ensure adequate coverage and support for study participants across the expanded study area.

##### Aligning dates for screening and enrollment

This adaptation involved synchronizing the screening and enrollment processes to occur on the same day for patients, aiming to alleviate travel and cost inconveniences for study participants. Initially, PLHIV underwent screening on one day, and if eligible for the TASSH study, they were enrolled on a separate day, typically 3 to 5 days later. However, during the implementation phase, the study team opted to adjust this approach. The modification loosened and streamlined the structure of the enrollment process to minimize barriers for participants. This decision was prompted by the observed low follow-up rate, attributed to the costs and inconveniences associated with multiple visits. By aligning the screening and enrollment dates, the goal of the modification was to boost both enrollment and retention rates by simplifying the process for participants, thereby enhancing their engagement with the study.

#### Enhancing patient follow-up and retention rates

##### Patients incentives to attend follow-up appointments

The study introduced incentives for patients to attend follow-up appointments, aiming to address low follow-up and retention rates among study participants. This adaptation involved providing transport vouchers to facilitate participants' attendance, acknowledging the significant distances between PHCs and their residences. Initially, the project implementation did not incorporate such incentives, and participants were scheduled for follow-up appointments without transportation assistance. However, recognizing the barriers posed by transportation issues, especially for patients with conditions like HIV or hypertension, who often face financial constraints, the modification was deemed necessary to improve retention rates. Implementing this modification aimed to enhance participants’ engagement and improve the study validity by addressing transportation challenges and financial constraints, thereby strengthening follow-up and retention rates within the study population.

##### At-home visits to deliver intervention

At-home visits to deliver the intervention were implemented to address the challenge of low follow-up and retention rates among participants. This adjustment involved nurses conducting visits to participants' homes to deliver the TASSH intervention, acknowledging the significant obstacle participants faced in traveling long distances to PHCs. Originally, participants were expected to visit PHCs per their scheduled follow-up window. However, due to slow follow-up and retention rates, attributed to patients' reluctance to seek hypertension care at PHCs, the modification became necessary to achieve the primary objective of the study. This adjustment tailored and refined the intervention delivery method to better meet participants' needs. By conducting at-home visits, the goal was to increase follow-up and retention rates, ensuring greater participant engagement and adherence to the study protocol.

##### Call log for tracking

The study introduced call logs to track reminders through phone calls for follow-up visits by nurses/CHOs to participants to address the low follow-up and retention rates. This adjustment involved nurses/CHOs implementing a structured call log to track the number of phone calls made to participants and their respective follow-up success rates. Although phone calls were made to participants initially, there was no systematic tracking or logging of their success rates. The modification introduced a structured call log to enhance tracking and monitoring efforts. This adaptation aimed to improve follow-up and retention rates by systematically tracking phone calls and their outcomes to increase the effectiveness of follow-up efforts, ultimately enhancing participant retention and engagement within the study.

##### Expanding window for allowable follow-up visits

Expanding the window for allowable follow-up visits aimed to improve retention by providing greater flexibility. This adaptation extended the follow-up window from ± 1 month to ± 3 months from the intended visit date. Initially, follow-up visits were planned at 6-, 12-, 18-, and 24-month intervals within a narrow one-month window. However, discussions with DSMB members revealed many patients fell outside this timeframe, prompting the modification during the implementation phase to allow more flexibility. This adjustment involved extending the pacing and timing of follow-up visits to retain more patient data, as advised by the DSMB. By expanding the allowable follow-up window, the goal was to increase retention rates by accommodating patients who might have otherwise missed their visits due to scheduling constraints or other factors.

#### Ensuring capacity building; ensuring the quality of intervention delivery; ensuring implementation fidelity

##### Increasing frequency of training

Increased frequency of TASSH training was implemented as a capacity-building strategy. This adaptation involved more frequent training sessions for all participating nurses/CHOs due to high turnover rates at clinics. Initially, training for participating nurses was scheduled when a new cohort was onboarded. However, in response to the high turnover, the training frequency was increased to ensure that new nurses/CHOs were updated on the TASSH protocol. This adjustment involved repeating elements or modules in the training sessions. The reason for this modification was the challenging circumstances contributing to high nurse turnover in Nigeria, such as rotations of nurses by the PHC board (across Lagos state) and opportunities for better pay or relocation of nurses abroad (brain drain). The goal of this modification was to ensure capacity building, maintain the quality of intervention delivery, and ensure implementation fidelity despite staff turnover.

Each of these adaptations mapped onto the FRAME modules are described in Table 1. The study noted that 75% (9) of the adaptations involved expanding the structure of the intervention and implementation strategies (marked as “adding elements”, “lengthening/extending” or “loosening structure”) and 50% (6) were implemented at the implementer level (as opposed to clinician, patient or organizational levels). Additionally, all adaptations were made during the implementation phase of the trial, were planned/reactive, and were mostly widespread to the implementation team (66.7%, 8). Additional details on each of the adaptations are found in Table 1.

**Table 1:**
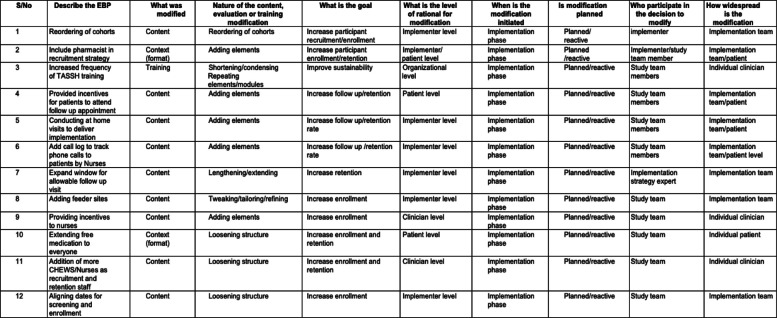
Adaptations of the trial using FRAME

**Fig. 3:**
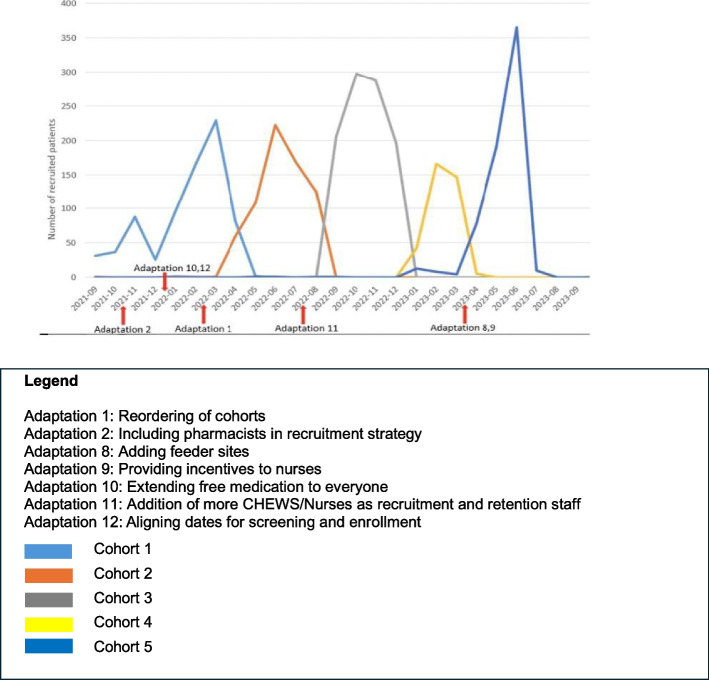
Monthly recruitment data by cohort

The study team monitored the effects of the adaptations for participants’ recruitment/enrollment by monitoring monthly recruitment figures. Figure 3 presents the monthly recruitment progress organized per cohort. Adaptations related to participant recruitment/enrollment (adaptations 1, 2, 8–12) were introduced throughout the study, and were followed by an increase in the number of recruited patients once implemented. This change was observed consistently across all cohorts, indicating that the implications of the adaptations yielded positive results for their purpose – increased recruitment.

## Discussion

In documenting the adaptations made to the TASSH study, we have highlighted the real-world complexities encountered during its implementation in PHCs across Lagos State, Nigeria. Our key findings identified twelve adaptations, each aimed at achieving one of three primary objectives: increasing participant enrollment and retention rates, ensuring capacity building, and enhancing both the quality of intervention and implementation fidelity. These adaptations were meticulously documented across eight domains within the FRAME model. Of all the adaptations, those focused on increasing participant recruitment had immediate positive impacts.

The most common adaptations in our study were made to the content of the intervention, with additional adjustments in the context and training categories. This finding aligns with previous studies, which have also reported a predominance of content adaptations [20–22]. However, our results differ from a study conducted in Kenya, where contextual adaptations were more prevalent [23]. This disparity may stem from the differing nature of the interventions. Our study focused on strengthening existing task-shifting policies for managing non-communicable diseases (hypertension) within the HIV population, whereas the Kenyan study centered on a phone-based delivery of an adolescent transition package for HIV-infected adolescents moving to adult care. The difference in target populations and intervention goals likely influenced the types of adaptations required in each study.

Due to the diversity in the PHC settings (size, operation hours, staff strength, and patient load), these adaptations were not uniformly applicable. Our adaptations were all reactive and occurred in the implementation phase of the primary study, with collective decisions (approval for adaptations) taken by the study team and project implementers. This is similar to a study conducted by *Schoenthaler, et al. *which documented adaptations to a multifaceted implementation strategy using FRAME-IS [24]. The Consolidated Framework for Implementation Research (CFIR) was utilized in the pre-implementation phase of both primary studies, however, the real-world settings not envisaged resulted in the challenges encountered during the implementation phase. This was aptly evident with the frequent turnover of trained healthcare workers (nurses, CHEWs and CHOs) due to staff transfers and relocation for greener pastures abroad.

The intersection of NCDs and infectious diseases—specifically hypertension and HIV in this context—continues to garner significant interest from healthcare providers, researchers, and policymakers. Providing concrete data to support the integration of these traditionally vertical healthcare systems is crucial for advancing sustainable health systems that prioritize the overall well-being of patients, moving beyond the current focus on specialized clinics. Despite challenges in realizing the chronic disease clinic model, its cost-effectiveness offers clear benefits for both healthcare providers and patients [25].

This study highlights the intricate dynamics between patients and healthcare workers that are often overlooked in routine epidemiological studies, particularly in the management of chronic diseases. These insights have equipped the study team with robust, pragmatic strategies essential for ensuring the intervention’s sustainability beyond the study period. The inclusion of a broader range of HCWs beyond the core focus on nurses and CHOs demonstrates a system-wide approach to addressing the challenges encountered. This approach fosters a collaborative ecosystem within the healthcare setting, benefiting not only the HCWs but also the patients and the wider community.

To our knowledge, this is the first study in our setting to document adaptations using the FRAME model to integrate hypertension management into HIV care in West Africa. FRAME was suitable in capturing general adaptations to evidence-based strategies, which are crucial for enhancing the adoption, sustainability, and scale-up of interventions, rather than focusing solely on evidence-based practices. Although the study was conducted within PHCs in Lagos State, our findings are applicable to PHCs across other states in Nigeria. This broader relevance is due to the Lagos State Primary Healthcare Board's pioneering approach to addressing staff shortages and its comparatively advanced health indicators. Moreover, the application of evidence-based practices in real-world healthcare settings demands thorough evaluations to determine their suitability for scaling up or for ensuring alignment within specific clinical environments. Adapting or modifying these evidence-based practices is essential for capitalizing on the lessons learnt and successes achieved during their implementation.

Despite the innovative potential of the FRAME model, several of its components were not applicable to our study. Additionally, some of the adaptations we implemented, such as out-of-facility or home visits, may not be easily replicable on a larger scale in real-world settings due to the significant financial resources and commitment required from HCWs. Moreover, the FRAME tool was not designed to measure the outcomes of documented adaptations, leaving a gap in understanding the impact of these changes. Lastly, the FRAME tool may not have fully captured all aspects of the TASSH intervention adaptations, potentially overlooking certain nuances or challenges related to fidelity.

In summary, documenting adaptations in implementation science studies is crucial for creating structured, insightful, and transparent pathways that enhance the scalability and sustainability of evidence-based practices.

## Conclusion

This study systematically documented and analyzed the adaptations implemented during the TASSH study (to integrate hypertension management into HIV care) at the PHCs in Lagos, Nigeria. By employing the FRAME model, the twelve identified adaptations addressed challenges in participants’ recruitment, retention, and intervention delivery, ultimately enhancing implementation fidelity. Our findings underscore the importance of flexibility and adaptability in complex public health interventions. By sharing these lessons, we aim to inform future research and implementation efforts in similar contexts, contributing to improved hypertension management within HIV care and broader primary healthcare systems.

## Supplementary Information

Additional file 1.

## Data Availability

Data for this article can be shared by the corresponding author upon reasonable request.

## Abbreviations

AAA: Angela A. Aifah
AB: Aderonke Bayonle
AOO: Aina Olufemi Odusola
ART: Antiretroviral Therapy
BOT: Bamidele Olusegun Tayo
CHN: Chioma Hope Nwankwo
CHOs: Community Health Officers
CHEWs: Community Health Extension Workers
DO: Deborah Onakomaiya
DO: David Oladele
DSMB: Data and Safety Monitoring Board
FRAME: Framework for Reporting Adaptations and Modifications for Evidence-Based Interventions
GO: Gbenga Ogedegbe
HCWs: Healthcare Workers
HIV: Human Immunodeficiency Virus
II: Ifeoma Idigbe
JH: Jiyuan Hu
JI: Juliet Iwelunmor
LGA: Local Government Area
LSACA: Lagos State AIDS Control Agency
LSPHCB: Lagos State Primary HealthCare Board
NCDs: Non-Communicable Diseases
NHLBI: National Heart, Lung, and Blood Institute
NIMR: Nigerian Institute of Medical Research
NIH: National Institutes of Health
NK: Nafesa Kanneh
ODO: Oluwayemi Dorcas Odejobi
OE: Oliver Ezechi
OOO: Oluwatosin Olaseni Odubela
PHC(s): Primary Healthcare Centers
PLWH: People Living with HIV
SM: Shivani Mishra
TASSH: Task Strengthening Strategy for Hypertension
UN: Ucheoma Nwasozuru
WC: Weixi Chen
ZM: Zaidat Musa
