# Supplementary material for: Adaptations to an implementation study for integrating hypertension management into HIV care in Lagos, Nigeria: application of the FRAME
Source: Implement Sci Commun. 2026 Mar 10;7:55. doi: 10.1186/s43058-026-00869-3 (PMC13032412; doi:10.1186/s43058-026-00869-3)
Supplement: Supplementary file 1 — Additional file 1. [file 43058_2026_869_MOESM1_ESM.docx]

Completed SQUIRE 2.0 Checklist for the manuscript titled **“Adaptations to an implementation study for integrating hypertension management into HIV care in Lagos, Nigeria: Application of the FRAME”**

## Title

Adaptations to an implementation study for integrating hypertension management into HIV care in Lagos, Nigeria: Application of the FRAME

## Abstract

The abstract provides details on the background, methods, results, and conclusion of the study. It summarizes the adaptations made to a randomized control trial (RCT) using the Framework for Reporting Adaptations and Modifications - Enhanced (FRAME) model.

## Problem Description

High dual-disease burden of hypertension and HIV in Nigeria requires an integrated healthcare approach. The trial aims to adapt and improve an evidence-based strategy to ensure that it is context specific, scalable, and sustainable.

## Available Knowledge

Existing literature highlights the need for context-specific task-shifting interventions in primary health clinics (PHCs) to integrate chronic disease management into HIV care.

## Rationale

The FRAME model was used to systematically document and analyze adaptations to the implementation strategy and interventions.

## Specific Aims

To characterize adaptations made to a late-stage implementation science trial integrating hypertension management into HIV care and their implications.

## Context

Study conducted in Lagos, Nigeria across 30 PHCs under a cluster RCT design.

## Intervention(s)

The TASSH intervention and Practice Facilitation implementation strategy were applied in the PHCs.

## Study of the Intervention(s)

The study used FRAME to document adaptations and their impact on implementation fidelity.

## Measures

Key measures included recruitment, retention rates, and intervention delivery fidelity.

## Analysis

A qualitative and quantitative analysis was conducted using triangulated data sources.

## Ethical Considerations

Ethical approval obtained from the implementation partner, the Nigerian Institute of Medical Research (NIMR) and NYU Grossman School of Medicine.

## Results

Twelve adaptations were made, categorized into recruitment, retention, and intervention fidelity improvements.

## Summary

Key findings demonstrate the need for flexible adaptation strategies in implementation trials.

## Interpretation

Findings align with previous studies on adaptation strategies but provide novel insights for low-resource settings.

## Limitations

Limited generalizability due to site-specific interventions; potential bias in adaptation documentation.

## Conclusions

The study highlights the importance of systematically documenting adaptations for future scale-up.

## Funding

Supported by a National Heart, Lung, and Blood Institute (NHLBI) of the National Institutes of Health (NIH) grant R01HL147811.
